# Familial Reversible Cerebral Vasoconstriction Syndrome: Insights From Two Families

**DOI:** 10.1155/crnm/3126513

**Published:** 2025-03-06

**Authors:** Pacôme Constant dit Beaufils, Benoît Guillon, Hugues Chabriat, Solène de Gaalon

**Affiliations:** ^1^Departments of Neurology, Nantes University Hospital, Nantes F-44000, France; ^2^Department of Neurology, Hôpital Lariboisière, Paris F-75010, France

**Keywords:** case report, familial, genetic, RCVS, reversible cerebral vasoconstriction syndrome, vasospasm

## Abstract

Reversible cerebral vasoconstriction syndrome (RCVS) is a rare condition whose exact underlying mechanisms remain undetermined. Herein, we report two exceptional family cases suggesting that a potential genetic factor might be involved in this condition. A mother and her daughter presented with recurrent thunderclap headaches and reversible vasoconstriction of the right middle cerebral artery in the first family. Clinical features suggestive of RCVS associated with a discrete subdural hemorrhage was observed in a mother and her daughter in a second family. These familial observations of RCVS suggest the existence of a genetic factor promoting the emergence of this condition.

## 1. Introduction

Reversible cerebral vasoconstriction syndrome (RCVS) is characterized by thunderclap headaches (TCHs) with reversible multifocal constriction of intracranial arteries. Various disturbances might promote to the emergence of this condition such as endothelial dysfunction, sympathetic hyperactivity, oxidative stress, blood–brain barrier disruption, or even some genetic predisposition to cerebrovascular autoregulation dysfunction [1]. Herein, we report two families in which a mother and daughter both had clinical and imaging manifestations of RCVS.

## 2. Case Presentation

### 2.1. Case 1 (Family 1)

**Figure 1:**
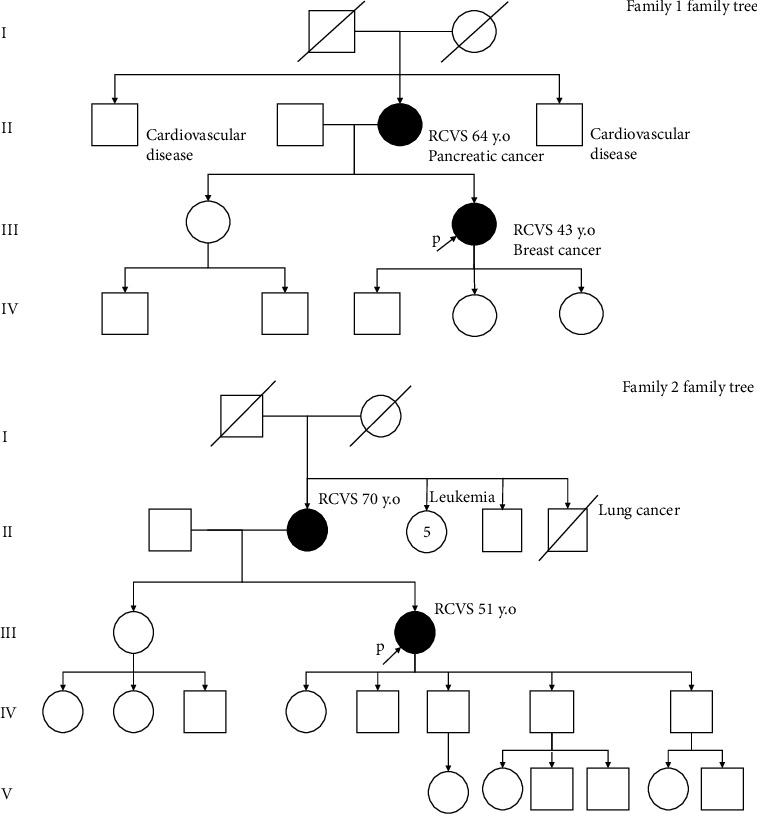
Family trees.

A 64-year-old woman, without any previous medical history or treatment, was admitted for TCH (Table 1). A cerebral CT angiography (CTA) revealed alternating constrictions and dilations of distal intracranial arteries especially on the right middle cerebral artery (MCA). The lumbar puncture showed normal cerebrospinal fluid (CSF) composition without xanthochromia. The patient was treated by nimodipine at Day 5 and did not complain of any recurrence of headache. CTA at 3 months was normal (Figure 1).

### 2.2. Case 2 (Family 1)

A 43-year-old woman with a past history of untreated migraine was hospitalized four years after her mother for TCHs. Two CTAs at Day 16 and Day 19 from clinical onset and one MRI at Day 22 were normal but transcranial doppler ultrasound (TCD) examination at Day 16 showed focal acceleration on the right MCA (151 cm·s^−1^ at M1 segment). Nimodipine was started at Day 17 and abstaining from sexual activity was recommended. The TCD examination performed 3 months after discharge was normal.

### 2.3. Case 3 (Family 2)

A 70-year-old woman, with only a past history of nontreated hypercholesterolemia, was admitted after TCH. A first CTA obtained at Day 4 was normal. A second CTA performed at Day 9 was still normal. The CSF examination showed 190 red blood cells/mm^3^ and 9 white blood cells/mm^3^ without xanthochromia. The patient was discharged the same day with nimodipine. TCD examination at Day 11 and MRI at Day 12 were normal.

### 2.4. Case 4 (Family 2)

A 51-year-old woman was admitted 80 days after her mother for TCH. Previously, she was treated by a statin for hypercholesterolemia and used hormone replacement therapy for menopause but no antithrombotic therapy. She did not report any head trauma; her neurological examination was normal. The cerebral CTA performed at admission was normal but CSF examinations after two repeated nontraumatic lumbar punctures obtained within 12 hours after the headache episode also showed > 400 red blood cells/mm^3^ without any xanthochromia and decreased proteinorachia (1.08 g·L^−1^–0.27 g·L^−1^) and white cell count (20 cells/mm^3^–7 cells/mm^3^) consistent with an uncertain subarachnoid hemorrhage. A digital subtraction angiography showed no arterial malformation or vasospasm. TCD examination at Day 3 showed an increased systolic velocity (125 cm·s^−1^) at the right anterior cerebral artery. A treatment by nimodipine was then started. At Day 7, an MRI showed a right subdural hematoma and segmental vasoconstriction of both anterior cerebral arteries, of right posterior cerebral artery, and of the basilar trunk but no subarachnoid hemorrhage or intracranial hypotension. Thirteen weeks after her TCH, TCD and MRI examinations showed a complete normalization of cerebral arteries.

## 3. Discussion

The most original aspect of this report is, at our knowledge, the first observation of RCVS in two first-degree relatives within two distinct families: a mother and her daughter presented with TCH. Only in one case, vasoconstriction was not documented by imaging or ultrasound examination. However, the absence of visible narrowing is possible in RCVS since the vasoconstriction is transient, may vary over time, in different vascular territories or remain limited or too distal. In this case, TCD may be useful noninvasive imaging modality to catch the dynamic nature of RCVS. Multimodal imaging is often required to diagnose the RCVS for particularly illustrating the centripetal widespread of vasoconstriction. This diagnosis should not be ruled out if this examination is normal, as it may reveal late abnormalities when repeated. Other examinations may also reveal functional disturbances in the vessels that are suggestive but delayed in time [2, 3]. Only in one case, focal vasoconstriction of intracranial arteries was not documented by imaging or ultrasound examination. However, the absence of visible stenosis is possible in RCVS since the vascular constriction is transient, may vary over time, in different vascular territories or remain limited or too distal [4, 5]. In this case, TCD may be useful noninvasive imaging modality to catch the dynamic nature of RCVS [6]. Distinguish headaches associated with sexual activity (HSA) and probable RCVS is always a challenge since imaging could be normal at the early stage of RCVS. A recent study of HSA using this semiology to distinguish HSA from probable RCVS showed a more prevalent recurrent sex headaches due to RCVS [7] Recurrence of TCH and typical triggers should lead to the diagnosis of RCVS, as recommended by ICHD-3, especially when brain imaging allows to rule out intracranial lesions [8]. Despite the lack of evidence, all patients received nimodipine that may reduce the course of the disease [9]. However, we cannot exclude potential effects related to trigger withdrawn as rest in the present cases.

The observations, which concern only women with similar benign phenotypes, which occurred without any chronological link in both cases, further reinforce the hypothesis of a genetic factor of susceptibility underlying RCVS. No other vascular abnormalities were detected in each case on the cervical artery. It has been hypothesized that patients with RCVS could have a genetically driven impairment of the cerebral vasoreactivity and that environmental factors such as acute stress or drugs use could act as precipitating factors [1]. Rarely, RCVS is associated with a known monogenic disease, such as vascular Ehlers-Danlos [10] or Loeys-Ditz syndrome [11] (Supporting information (available here)).

Patients with RCVS also often present concomitant arterial anomalies such as persistent autonomic dysreflexia [12], endothelial dysfunction [13], cerebral autoregulation impairment [14], and an association with intracranial aneurysms [15]. A different characteristic' and patient's outcome has been suggested by some authors according the ethnicity [5]. Another argument suggesting a genetic predisposition is the co-occurence of carotid artery dissection in 10% of RCVS [16], a disease where also sporadic cases have connective tissue abnormalities [17] and a high prevalence of polymorphism in PHACTR1 gene [18]. Progress in unraveling the genetics of RCVS will require the collection of genetic samples from large multicenter series as proposed by the REVERCE consortium [19]. In the literature, only few data suggest a potential genetic predisposition to vasospasm after subarachnoid hemorrhage and some variants have been detected associated with the occurrence of vasospasm (eNOS, Hp, ApoE, CBS, ENDRA, and SERPINE1) or with its severity (eNOS, PAI-1, ApoE, RyR1, AT2, DDHA1, HMGB1, and ACE) [20, 21].

Finally, our observation suggests that a particular attention should be paid to look for other intrafamilial observations of RCVS and that future investigations may be needed to confirm the existence of a genetic factor promoting the emergence of this transient vascular storm that remains of undetermined origin but with a lot of environmental triggers.

## Figures and Tables

**Table 1:** Main characteristics of patients.

Case presentation	Family 1		Family 2	
	1	2	3	4
Headache characteristics	Right-sided hemicrania	Right-sided occipital headache	Holocranial headache	N/A
Number of headache	1 TCH	> 10 TCH (1–4/d) over 16 days	3 TCH (1/d) over 6 days	1 TCH
Duration	60 min	1–60 min	60–90 min	< 15 min
Trigger	None (physiological activity)	Sexual activity	Acute emotional situation/Bathing	None (rest on sofa)
Precipitant factors	No	No	No	No
Blood pressure (cmHg)	12/7	12/6	12/7	11/8
Neurological examination	Normal	Distal motor deficit of her left upper limb	Normal	Normal
Vasospasm imaging modality	CTA	TCD	N/A	MRA
Nimodipine (oral administration)	60 mg, 4 times per day, during 30 days	60 mg, 6 times per day, during 20 days	30 mg, 4 times per day	60 mg, 4 times per day
Diagnosis according ICHD-3	6.7.3.1 acute headache attributed to RCVS	6.7.3.2 acute headache probably attributed to RCVS	6.7.3.2 acute headache probably attributed to RCVS	6.7.3.1 acute headache attributed to RCVS
Recurrence/follow-up (years)	None/5	None/3	None/8	None/8

*Note:* ICHD-3, third edition of the international classification of headache disorders.

Abbreviations: CTA = computed tomography angiography, d = day, MRA = magnetic resonance angiography, N/A = not available, RCVS = reversible cerebral vasoconstriction syndrome, TCD = transcranial Doppler, TCH = thunderclap headaches.

## Data Availability

Data sharing is not applicable to this article.

## Nomenclature

RCVS: Reversible cerebral vasoconstriction syndrome
TCHs: Thunderclap headaches
CTA: Computed tomography angiography
MCA: Middle cerebral artery
TCD: Transcranial Doppler
SAH: Subarachnoid hemorrhage
HAS: Headaches associated with sexual activity
MRI: Magnetic resonance imaging
MRA: Magnetic resonance angiography
CSF: Cerebrospinal fluid
ICHD-3: Third edition of the international classification of headache disorders

## Supporting Information

Summary of previous RCVS cases with Mendelian disease.
