# Supplementary material for: Familial Reversible Cerebral Vasoconstriction Syndrome: Insights From Two Families
Source: Case Rep Neurol Med. 2025 Mar 6;2025:3126513. doi: 10.1155/crnm/3126513 (PMC11991833; doi:10.1155/crnm/3126513)
Supplement: Supporting Information — Additional supporting information can be found online in the Supporting Information section. [file 3126513.f1.docx]

**Supplemental material**: Summary of previous RCVS cases with Mendelian disease

| Case | Case 1[7] | Case 2[8] |
| --- | --- | --- |
| Sexe/Age (years) | Male/ 27 | Male/9 |
| Family history of RCVS | No | No |
| First symptoms | Severe headaches | Sudden severe headaches |
| Vessel anomaly | Aneurysm of the internal carotid artery | Arterial redundancies |
|  | Fusiform dilatation of the right anterior cerebral artery, irregular middle cerebral arteries bilaterally with focal dilatation at the right M1-M2 junction and bilateral narrowing of the proximal M2 arteries. The P3 segments of the posterior cerebral artery had bilateral stenoses | Mild narrowing of the bilateral internal carotid arteries |
| Concurrent dissection | Yes | Yes |
| Underlying genetic predisposition | Ehlers-Danlos syndrome, type IV | Loeys-Dietz syndrome |
| Complication | No | PRES, SAH |

RCVS, reversible cerebral vasoconstriction syndrome ; PRES, posterior reversible encephalopathy syndrome ; SAH, subarachnoid hemorrhage.
